# Personal News Items

**Published:** 1915-05

**Authors:** 


### Personal News Items.

Drs. J. F. Mulkey and W. A. Bishop were recently elected members of the city board of education of Kaufman, Texas.

Dr. L. R. Talley, of Temple, has returned after spending several months in post-graduate work in the North and East.

Dr. Thomas V. Fryar, of Dallas, has been appointed chief surgeon of the Texas National Guard, to succeed A. B. Kennedy, of Bonham.

The friends of Dr. C. M. McCollum will be gratified to learn that he has been tendered and has accepted a chair in the Fort Worth Medical College.

Dr. M. M. Carrick has been called from New York because of the serious illness of his mother. He has the sympathy of his many friends in Texas.

Dr. S. W. Rimmer, of San Saba, left home on the 8th to join his wife at the bedside of her mother, Mrs. Mary Bewley, of Austin, who is seriously ill.

Dr. G. E. Dickinson has returned to his home in San Benito after an absence of two months spent at Mineral Wells. He has again resumed his practice.

Dr. J. M. Eastland, of Mineral Wells, was elected President of the Northwest Texas Medical Society at its forty-third semi-annual meeting held in Wichita Falls the 13-14th inst.

Dr. J. D. Michie, of Childress, has again been elected county health officer, a position he has held with much honor. His appointment will be met with much favor.

Dr. I. L. Maxwell, of Wylie, while beating a carpet with a strap, received a very painful and what may prove to be a very serious injury by a part of the strap striking him in the eye.

---

### Change of Location.

Dr. Nelson, of Cumby, is now located at Frost.

Dr. J. B. Ramsey, of Alto, is now located at Forest, Texas.

Dr. H. A. Briggs, of Palestine, has moved to Buffalo, Texas.

Dr. Stringer has recently moved from Oklahoma to Dexter, Texas.

Dr. J. H. Barnett, of De Leon, is now located at Walnut Springs.

Dr. W. L. McNail and family, of Arlington, have moved to Graham.

Dr. J. H. Petty has recently moved from Taylor to San Antonio, Texas.

Dr. Wm. Stanley is now located at Cat Springs. He formerly lived in San Antonio.

Dr. Wheeler has moved from Christine to Campbellton.

Dr. Lightfoot, who takes the place of Dr. Cockrell, is now located at Blossom.

Dr. Harry Elwood Hoke, of McLennan county, who is associated with Dr. Wood in the Hubbard City Sanitarium, has registered for the practice of medicine and surgery in Hillsboro.

